# Transgenerational Adaptation of *Arabidopsis* to Stress Requires DNA Methylation and the Function of Dicer-Like Proteins

**DOI:** 10.1371/journal.pone.0009514

**Published:** 2010-03-03

**Authors:** Alex Boyko, Todd Blevins, Youli Yao, Andrey Golubov, Andriy Bilichak, Yaroslav Ilnytskyy, Jens Hollander, Frederick Meins, Igor Kovalchuk

**Affiliations:** 1 Department of Biological Sciences, University of Lethbridge, Lethbridge, Canada; 2 Friedrich Miescher Institute for Biomedical Research, Basel, Switzerland; 3 Department of Plant Systems Biology, VIB, Ghent University, Ghent, Belgium; 4 Department of Molecular Genetics, Ghent University, Ghent, Belgium; Michigan State University, United States of America

## Abstract

Epigenetic states and certain environmental responses in mammals and seed plants can persist in the next sexual generation. These transgenerational effects have potential adaptative significance as well as medical and agronomic ramifications. Recent evidence suggests that some abiotic and biotic stress responses of plants are transgenerational. For example, viral infection of tobacco plants and exposure of *Arabidopsis thaliana* plants to UVC and flagellin can induce transgenerational increases in homologous recombination frequency (HRF). Here we show that exposure of *Arabidopsis* plants to stresses, including salt, UVC, cold, heat and flood, resulted in a higher HRF, increased global genome methylation, and higher tolerance to stress in the untreated progeny. This transgenerational effect did not, however, persist in successive generations. Treatment of the progeny of stressed plants with 5-azacytidine was shown to decrease global genomic methylation and enhance stress tolerance. *Dicer-like* (*DCL*) *2* and *DCL3* encode Dicer activities important for small RNA-dependent gene silencing. Stress-induced HRF and DNA methylation were impaired in *dcl2* and *dcl3* deficiency mutants, while in *dcl2* mutants, only stress-induced stress tolerance was impaired. Our results are consistent with the hypothesis that stress-induced transgenerational responses in *Arabidopsis* depend on altered DNA methylation and smRNA silencing pathways.

## Introduction

Changes in epigenetic regulation of gene expression induced by environmental exposure can persist in the next sexual generation in stressed animals and plants [1], [2], [3], [4]. In some cases, these transgenerational effects can even be inherited over successive generations [3].

Seed plants can rapidly adapt in their response to abiotic and biotic stresses [5], [6], [7]. One mechanism of stress tolerance–acclimation–is characterized by the ability of the plant to change its physiology in such a way that stress does less damage [3], [7], [8]. Exposure to stress can also lead to genome instability and changes in DNA methylation [9], [10], [11]. Our earlier studies of *Arabidopsis* and *Pinus silvestris* growing in the vicinity of the Chernobyl reactor suggested that increased global methylation of the genome is correlated with genome stability and stress tolerance in response to irradiation[12], [13].

Transgenerational transmission of changes in homologous recombination frequency (HRF) has been reported recently for stressed *Nicotiana tabacum* and *Arabidopsis* plants. We showed that the progeny of tobacco plants infected with tobacco mosaic virus exhibited a high frequency of rearrangements at disease resistance gene-like loci, global genome hypermethylation, and locus-specific hypomethylation [14]. Based on studies with transgenic Arabidopsis lines carrying a β-glucuronidase (GUS) gene-based substrate for homologous recombination, Molinier et al. (2006) reported increased somatic recombination in progeny of plants exposed to UVC and to the bacterial elicitor flagellin. Moreover, the increased HRF triggered by UVC persisted for five subsequent untreated generations [3]. In contrast, more recent studies have led to the conclusion that transgenerational transmission of enhanced HRF in the same reporter lines is somewhat sporadic and limited to just four of ten stress conditions tested [15]. Because germ cells develop during stress treatment, changes that persist in the next generation can be referred to as either germline effects or transgenerational effects. To be consistent with recent publications [3], [15], we refer to these changes as transgenerational effects.

The available evidence suggests that plants have the potential for limited transgenerational transmission of changes in HRF in response to stress. Here we show that exposure of *Arabidopsis* plants to various abiotic stresses results in substantial transgenerational increases in the frequency of HFR, higher tolerance to stress, and global hypermethylation of the genome. These changes were not maintained in successive generations in the absence of stress. Stress tolerance depended on changes in the genome methylation and *Dicer-like* (*DCL*) *2* and *DCL3* which encode Dicer activities important for small RNA pathways implicated in epigenetic regulation.

## Results

### Measurement of Transgenerational Responses to Stress

We used transgenic *Arabidopsis* plants carrying either β-glucuronidase (GUS) or luciferase (LUC) recombination reporters to quantify transgenerational effects of stress on HFR.

**Figure 1:**
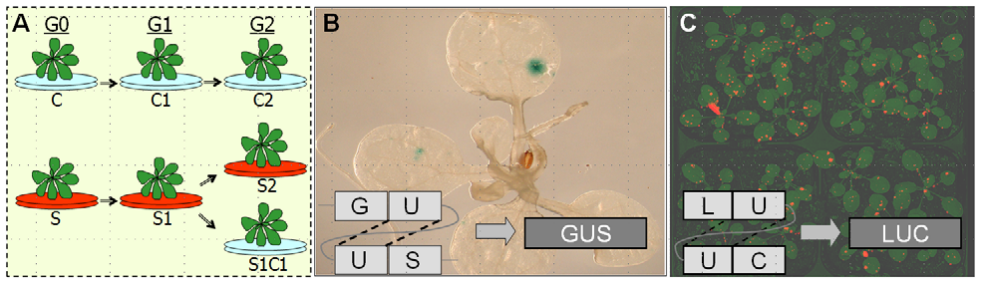
Experimental set-up. **A**. *Arabidopsis* plants (G0) were propagated to the next generation (G1) under normal growth conditions (C1) or in the presence of stress (S1 for ‘stressed, generation 1’). Next, the S1 plants were propagated to G2 in the presence of stress (S2) or under normal conditions (S1C1). The C1 plants were propagated to G2 under normal conditions (C2). **B–C**. Plants used in the experiment carried in the genome β-glucuronidase (GUS) or luciferase transgenic marker genes serving as a homologous recombination substrate. Double strand break in the region of homology (depicted as ‘U’) can potentially be repaired via homologous recombination using the second region of homology as a template. This restores the active transgene. Cells and their progeny in which recombination events occurred can be visualized via either histochemical staining (GUS) (**B**) or via CCD camera (LUC) (**C**). Individual events are then scored in the population of 20–200 plants and expressed as an average number per single plant.

The influence of salt stress was analyzed using the GUS transgenic line ll [16], and the influence of drought, flood, heat, cold and UVC stresses was analyzed using the LUC transgenic line 15d8 [17]. Treated plants did not show conspicuous developmental abnormalities under stress conditions that we used. Stressed (S) and untreated control (C) plants were self-fertilized to generate S1 and C1 plants, respectively (Figure 1A). C1 plants grown under control conditions were selfed to generate C2 plants; S1 plants grown under stress or control conditions were selfed to generate S2 and S1C1 plants, respectively (Figure 1A). The frequency of homologous recombination was estimated by measuring the incidence of blue spots caused by restoration of GUS activity in plants from line 11 and by analyzing light generated by restoration of LUC activity in plants from line 15d8 (ref. [17]) (Figure 1B,C). Stress adaptation was estimated by comparing relative growth of stressed and untreated plants.

### Progeny of Stressed Plants Exhibit Increased Frequencies of Homologous Recombination under Non-Stress Conditions

**Figure 2:**
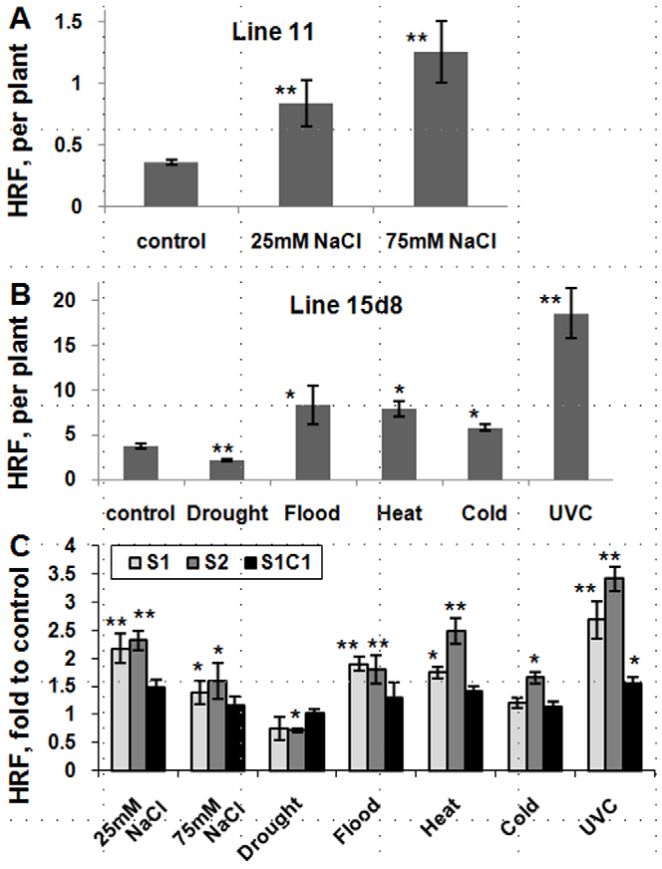
*Arabidopsis* plants show changes in somatic and transgenerational homologous recombination frequency (HRF) in response to stress. HR events were counted in transgenic *Arabidopsis* plants from line 11 exposed to NaCl and from line 15d8 exposed to heat, cold, drought, flood and UVC. Asterisks show significant differences relative to controls, where one is *p*<0.05 and two is *p*<0.01 (a single- factor ANOVA). **A.** Somatic HRF is shown as the average number of events per single plant (the average of three experiments and s.d.) in a population of 200 plants per experimental group. **B.** Somatic HRF is shown as the average number of events per single plant (the average of three experiments and s.d.) in a population of 50 plants per experimental group. **C.** Non-induced HRF (the average of three experiments and s.e.m., as calculated from 50 plants per each experimental group) in the S1, S2 and S1C1 plants grown under control conditions. The data are shown as fold of respective to the control (C1 and C2) for the plants exposed to NaCl, drought and flood, heat and cold, and UVC.

Exposure to salt, flood, heat, cold and UVC stresses significantly (*p*<0.05) increased HRF by 2-6-fold relative to control plants (Figure 2A,B). In contrast, exposure to drought decreased HFR. The S1 generation obtained by selfing salt-, heat-, cold-, UVC- and flood-stressed plants consistently exhibited a significant (*p*<0.05) increase in HRF relative to the C1 controls when plants were assayed under control conditions (Figure 2C; Table S1). In contrast, under the same conditions, the drought-stressed S1 plants exhibited a decrease in HRF relative to the C1 controls. These results indicated that stress treatment of parental plants leads to transgenerational changes in HRF.

To examine the inheritance of this transgenerational effect, we compared HRF of next-generation progeny plants after two generations of stress (S2 plants) and after one generation of stress followed by one generation of control treatment (S1C1). The results showed that whereas changes in HRF usually persisted in the S2 generation plants, the S1C1 plants usually exhibited HRF values similar to control (Figure 2C; Table S1). Thus, with the exception of a slight but significant effect in UVC-treated plants and plants exposed to 25 mM of NaCl, the transgenerational effect does not appear to be persistent in successive generations of untreated plants.

### Progeny of Salt-Stressed Plants Show Enhanced Adaptation to Stress

**Figure 3:**
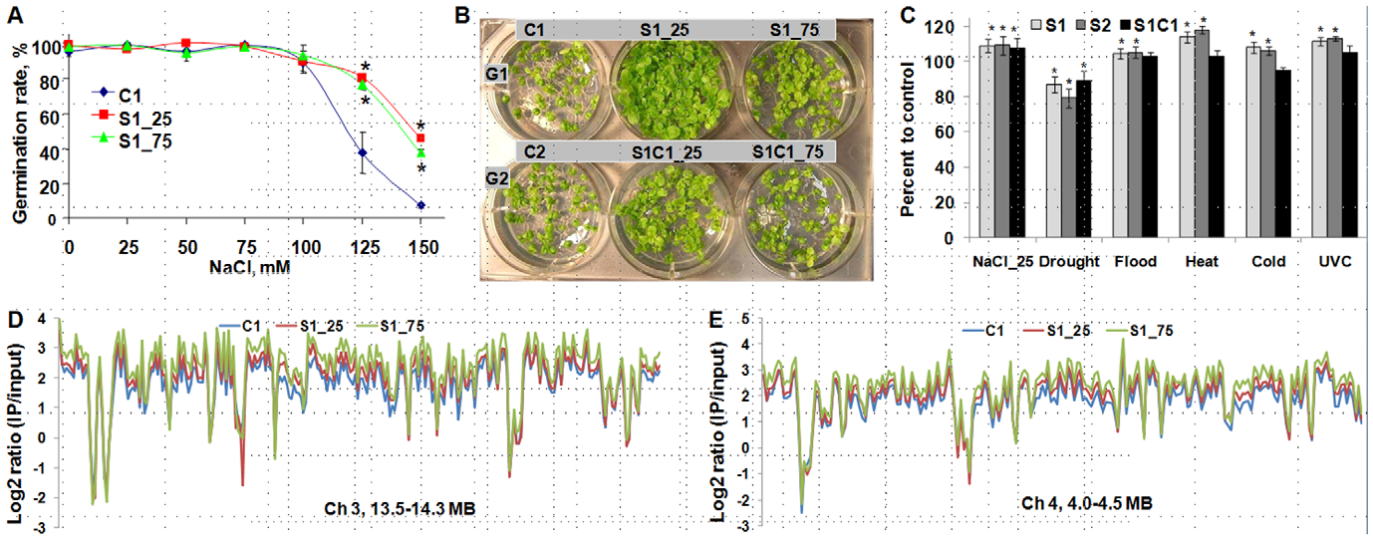
Progeny of salt-stressed plants exhibit higher tolerance to salt and changes in methylation pattern. **A.** NaCl tolerance was evaluated by germinating the progeny of plants exposed to 25 (S1_25) and 75 (S1_75) mM NaCl on media supplemented with 0–150 mM NaCl. Germination rates are shown in percentage (the average of three experiments and s.e.m., as calculated from 100 plants per plate, three plates per each experimental group). Asterisks show significant differences relative to controls (*p*<0.05, a single-factor ANOVA). **B.** The G1 and G2 (Figure 1A) generations of control and stressed plants were used for the analysis of tolerance to 150 mM NaCl. The S1 and S1C1 plants stemming from exposure to 25 and 75 mM NaCl are labeled as S1_25, S1_75 and S1C1_25, S1C1_75. Thirty to forty plants per each experimental group were geminated on normal media and then transferred to 150 mM NaCl. The picture was taken after two weeks of exposure. **C.** Global genome methylation patterns in the progeny of plants exposed to NaCl, drought, flood were analyzed using a cytosine extension assay [14]. Methylation levels (the average of three experiments ± s.d.) are shown relative to the control groups (100%) (C1 or C2). Asterisks show significant differences relative to controls, where one is *p*<0.05 and two is *p*<0.01 (a single- factor ANOVA). **D.** MeDIP analysis of methylation in C1, S1_25 and S1_75 plants. The figure shows the methylation level as reflected by a log_2_ ratio of intensities of immunoprecipitated to input DNA in the region of 13.5–14.3 MB of the centromeric area of chromosome 3. Data for the C1 is in blue, whereas data for S1_25 and S_75 are in red and green, respectively. Data show hypermethylation of centromeric areas at chromosome 3 of the S1_25 and S1_75 plants. **E.** MeDIP analysis of methylation in C1, S1_25 and S1_75 plants. The figure shows the methylation level as reflected by a log_2_ ratio of intensities of immunoprecipitated to input DNA in the region of 4.0–4.5 MB of the centromeric area of chromosome 4. Data for the C1 is in blue, whereas data for S1_25 and S_75 are in red and green, respectively. Data show hypermethylation of centromeric areas of chromosome 4 of the S1_25 and S1_75 plants.

Cold and heat stress is known to result in adaptation to stress, which in some cases is transmitted to progeny of stressed plants [5], [6], [18]. To determine if the stress-induced transgenerational changes in HRF that we observed are associated with stress adaptation, we treated the same GUS-reporter line with NaCl, and examined the germination rate and growth of progeny raised on increasing concentrations of NaCl. The progeny of plants exposed to 25 mM NaCl and 75 mM NaCl showed a significantly higher (*p*<0.001 and *p*<0.01) germination rate when raised on 125–150 mM NaCl than did the progeny of untreated C1 plants (Figure 3A). The growth of the progeny of salt-stressed plants (S1) was enhanced at 150 mM NaCl relative to the progeny of controls (Figure 3B). In contrast, growth was only slightly enhanced in S1C1 plants relative to controls. These results show that salt-induced salt adaptation and salt-induced increases in HRF are transgenerational effects that generally do not persist in successive generations. We also tested how the progeny of plants exposed to salt (S1_25 and S1_75) and the progeny of control plants (C1) respond to methyl methane sulfonate (MMS). We found that both S1_25 and S1_75 were partially tolerant to MMS (Figure S1). MMS is a genotoxic agent commonly used for analysis of stress tolerance in various DNA repair and genome stability mutants [19]. It is a DNA-methylating agent predominantly resulting in 7-methylguanine (N7-MeG; 82% of all types of damages) and inducing sister chromatid exchanges as well [20], [21]. It is believed that a MMS-dependent increase in recombination frequency is induced by base excision repair-generated strand break intermediates [20].

### Progeny of Stressed Plants Exhibit Changes in Global DNA Methylation under Non-Stressed Conditions

Transgenerational effects in both plants and animals are often associated with alterations in methylation of genomic DNA [1], [2], [22]. This prompted us to compare the 5-methylcytosine (5-MeC) content of genomic DNA isolated from progeny of stressed and control plants. Methylation was analyzed in progeny plants germinated and grown under non-stressed conditions. Relative to the progeny of control plants of the same generation, the progeny of plants subjected to salt, flood, heat, cold and UVC stresses exhibited the significant (*p*<0.05) ca. 10–12% increases in the 5-meC content in S1 and S2 (Figure 3C). In contrast, drought-stressed plants exhibited a significant (*p*<0.05) ca. 15% decrease in the 5-meC content relative to controls. No significant differences were observed between the S1 and S2 plants, suggesting that prolonged stress for an additional generation does not increase DNA methylation. DNA methylation changes in the S1C1 plants remained similar to the S1 and S2 plants in the progeny of NaCl- and drought-exposed plants. In contrast, DNA methylation of the S1C1 plants stemming from other stress exposures did not differ from that of the C2 plants, suggesting that if stress is not maintained, DNA methylation tends to decrease (Figure 2C).

To find out whether changes in HRF in the transgene correlate with changes in methylation, we performed bisulfite sequencing of C1 and S1_25 plants. We found that the methylation level of 130 bp of the 35S promoter region was similar in both C1 and S1_25 plants (Figure S2). This could possibly suggest that changes in HRF in the transgene locus do not correlate directly with methylation levels at the promoter region.

**Figure 4:**
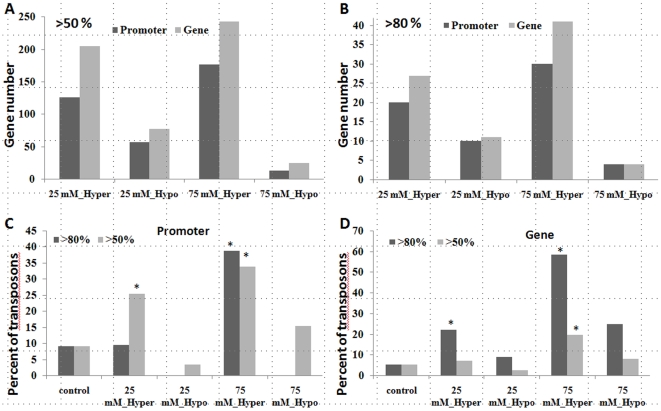
Analysis of methylation using Nimblgen tiling arrays shows many hypermethylated genes and promoters. Methylation levels at a 5 kb promoter region and at a transcribed region of a gene were compared between S1_25 and C1 groups as well as between S1_75 and C1 groups. Regions with methylation changes of more than 50% and 80% were identified. Figure shows the number of genes and promoters that exhibit either more than 50% (**A**) or 80% (**B**) of methylation changes in S1_25 and S1_75 plants as compared to C1 plants. Figure **C** shows the percentage of transposons among all genes that were hyper- or hypomethylated at the promoter in S1_25 and S_75 plants. Figure **D** shows the same for the transcribed region.

In more detailed studies, we measured global genome methylation of chromosomes 2, 3 and 4 by methylated DNA immuneprecipitation (MeDIP) using Nimblegene array #2. The centromeric region of chromosomes 3 and 4 was substantially hypermethylated in the progeny of plants exposed to 25 or 75 mM NaCl (Figure 3D,E). In contrast, several other regions of chromosomes 2, 3 and 4 were hypomethylated (Figure S3). We also compared the methylation status of genic regions of chromosomes 3 and 4. For the analysis, we used the 5 kb sequence 5′ of a transcribed region defined as promoter region and the transcribed sequence itself. We compared differences in methylation between S1_25 and C1 as well as S1_75 and C1 plants. Regions in S1_25 and S_75 plants were scored as hypermethylated if 50% and 80% higher levels of methylation as compared to C1 plants were reported, and they were scored as hypomethylated if 50% and 80% lower levels of methylation were detected. The analysis showed that S1_25 plants had twice as many hypermethylated promoters and transcribed regions than hypomethylated ones, whereas in S1_75 plants the number of hypermethylated genic regions was 10-fold more (Figure 4A,B). Among hypermethylated genes, there were transposable element-related genes (Figure 4C,D), genes involved in signaling, transcription, protein metabolism, histone modifications, stress and pathogen response (Table S2); whereas among hypomethylated genes, we observed overrepresentation of genes involved in signaling and DNA repair (Table S3).

These results are consistent with our measurements of the total meC content and show that despite global genome hypermethylation in response to stresses, many loci in the genome are hypomethylated.

### Progeny of Stressed Plants Show Substantial Changes in Global Gene Expression

**Figure 5:**
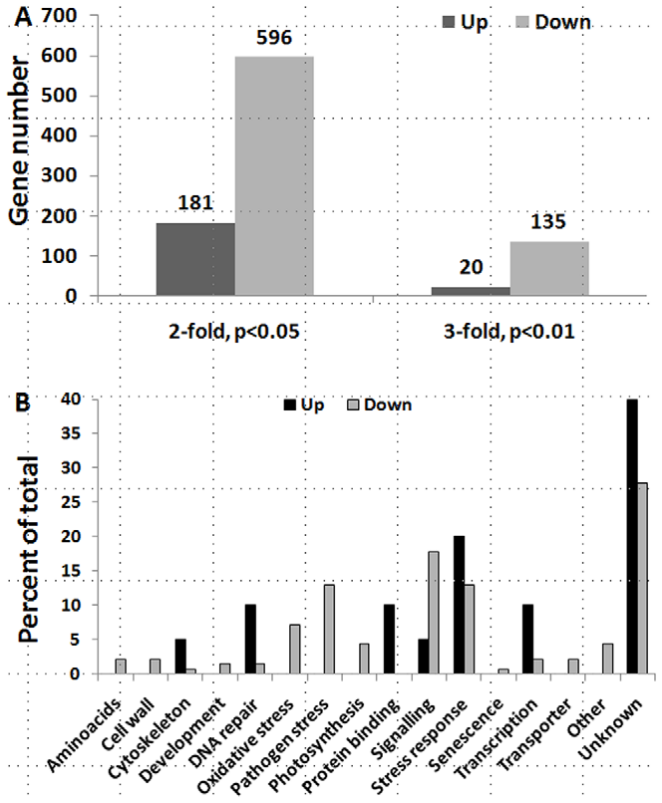
S1_25 plants differ from C1 plants in expression of many genes. Analysis of gene expression in S1_25 and C1 plants was done using Affymetrix microchips. The data from 3 chips per each experimental group were averaged, and two different cut-offs were performed. One was a 2-fold change and p-value of less than 0.05, and another–a 3-fold change and p-value of less than 0.01. **A.** Figure shows the number of up- and down-regulated genes belonging to the S1_25 group as compared to the C1 group. The numbers over the top of the bars show the gene number. **B.** Figure shows the percentile distribution of up- and down-regulated genes belonging to different pathways.

We used microchip analysis to detect changes in the transcriptome associated with stress-treatment of parent plants. We compared the transcriptome of S_25 and C1 plants using three independent biological repeats for each experimental group. S1_25 plants exhibited substantial changes in gene expression relative to control C1 plants. Using two-fold changes in expression and *p*<0.05 as a criterion, 181 genes were up-regulated and 506 were down-regulated at the RNA level (Figure 5A). Using stringent criteria, three-fold changes in expression and *p*<0.01, we identified 20 up-regulated genes and 135 down-regulated genes (Figure 5A). The majority of these genes (85%) were down-regulated. Numerous genes involved in abiotic and pathogen stress responses and signaling were included in this group (Table S4). Genes involved in pathogen response represented 13% of all down-regulated genes; none of these genes were up-regulated. Moreover, even using 2-fold changes and *p*<0.05, less than 2% of pathogen response genes exhibiting a 2-fold change (*p*<0.05) were up-regulated. Genes involved in transcription and genes involved in DNA repair represented 10% of all up-regulated genes; less than 2% of genes in either of the categories were down-regulated.

We confirmed by semi-quantitative RT-PCR the expression level changes of 6 selected genes with changes in expression over 5-fold (*p*<0.01). Within this set, 2 genes were up-regulated and 4 genes were down-regulated. RT-PCR confirmed the trend for all six selected genes (Figure S4).

### 5-azaC Treatment Blocks both NaCl-Induced Salt Tolerance and Hypermethylation of DNA

**Figure 6:**
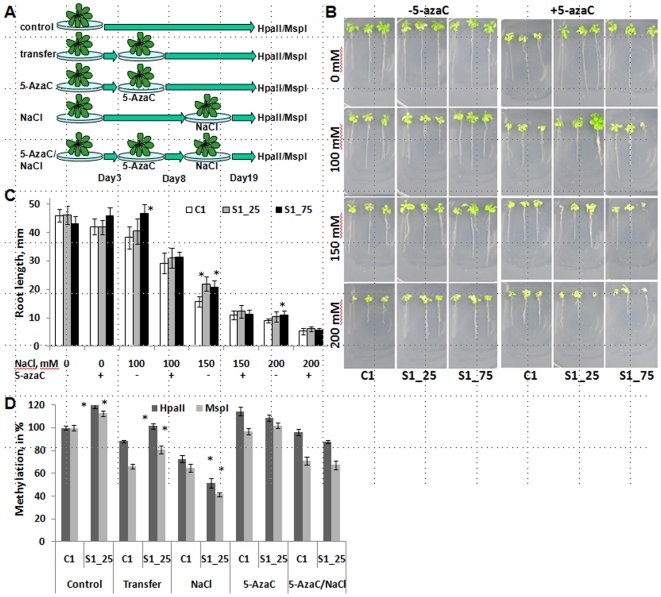
Pre-treatment with 5-azaC alleviates differences in stress tolerance and methylation changes. **A.** Twenty plants per each experimental group were germinated in half-MS medium. The plants of control group remained in this medium for the entire length of the experiment. At 3 dpg, the plants belonging to a ‘transfer’ group were transferred to similar half-MS medium and served as a ‘transfer’ control. At 3 dpg, the plants of the ‘5-AzaC’ group were transferred to 50 µM 5-azaC. At 3 dpg, the plants of the ‘5-AzaC/NaCl’ group were transferred to 50 µM 5-azaC, and at 8 dpg, they were transferred to 100 mM NaCl. At 8 dpg, the plants of the ‘NaCl’ group were transferred to 100 mM NaCl. All plants were harvested at 19 dpg, and genomic DNA was prepared and digested either with *Hpa*II or *Msp*I. The experiment was repeated three times. **B.** Twenty plants from Ct1, S1_25 and S1_75 groups were germinated on half-MS medium supplemented with or without 5-azaC and at the age of one week were moved to 0, 100, 150 and 200 mM NaCl. The experiment was repeated three times. **C.** Root length (the average of 3 independent plates, 20 plants per each plate, with s.e.m.). Asterisks show significant differences between the S1_25 and the C1 group and the S1_75 and the C1 groups (a single-factor ANOVA, *p*<0.05, for all groups). **D.** The data are shown as percentage of methylation related to the methylation level in the C1 plants of the control group. Significant differences between S1_25 and C1 for each group (p<0.05 in all cases) are labelled with asterisks.

To find whether higher tolerance to stress depends on DNA methylation, we treated the progeny of salt-stressed and control plants with 5-azacytidine (5-azaC), which is known to block methylation of cytosines in eukaroytes, including *Arabidopsis* [23]. Seeds were germinated on sterile media and transferred to media with and without 50 µM of 5-azaC 3-days-post germination (dpg). The control and 5-azaC plants were the transferred to media containing NaCl at 8 dpg to assay for tolerance to salt stress (Figure 6A).

First we analyzed stress tolerance by measuring root growth of 19-day-old plants. The results confirm that pretreatment of plants with NaCl increases tolerance of progeny to NaCl stress. This effect was most pronounced when pretreated plants were grown on medium supplemented with 150 or 200 mM (Figure 6B). The important point is that a significant effect of NaCl pre-treatment on salt tolerance was eliminated by pre-treatment of plants with 5-azaC (Figure 6B,C). Similar results were observed when plants pretreated with 5-azaC were exposed to methyl methane sulfonate (MMS) (Figure S5).

Next, we examined the relationship between 5-azaC-induced effects on salt-tolerance and DNA methylation. We analyzed methylation via the cytosine extension assay in DNA digested with *Hpa*II and *Msp*I, as restriction digestion with either of these enzymes is methylation-sensitive in plants. The analysis was performed in the S1_25 plants, since they showed stronger HRF changes and stress tolerance. Figure 6D confirms that the genome of S1_25 plants is hypermethylated as compared to the C1 plants. The experiment also showed that exposure of S1_25 plants only to NaCl (Figure 6A, ‘NaCl’) resulted in drastic hypomethylation, more pronounced in the S1_25 plants (Figure 6D). This suggests that despite genome-wide hypermethylation, the S1_25 plants respond to salt stress with more pronounced hypomethylation as compared to the C1 plants. Curiously, this decrease in methylation in response to NaCl was prevented in plants that were pretreated with 5-azaC (Figure 6D). Simply transferring plants from one control media to another also resulted in decreased DNA methylation (Figure 6D). The close correlation between loss of stress tolerance and DNA methylation in response to 5-azaC treatment supports the hypothesis that stress tolerance depends on alterations in DNA methylation.

### Transgenerational Effects on HRF, Stress Responses and DNA Methylation Are Affected in *dcl* Mutants

Our experiments suggest that exposure to stress results in changes in HRF and DNA methylation transmitted to the next generation. It has been proposed that changes in non-symmetrical DNA methylation could be maintained via the function of specific small interfering RNAs (siRNAs) [24]. Since the biogenesis of siRNAs and other related smRNAs depends on Dicer activities encoded by DCL1-DCL4 in Arabidopsis [25], we examined stress responses of the recombination reporter line 15d8 in plants homozygous for individual deficiency mutants *dcl2*, *dcl3, dcl4,* double *dcl2 dcl3* mutants, and triple *dcl2 dcl3 dcl4* mutants. The *dcl1* mutant lines were sterile and could not be tested.

**Figure 7:**
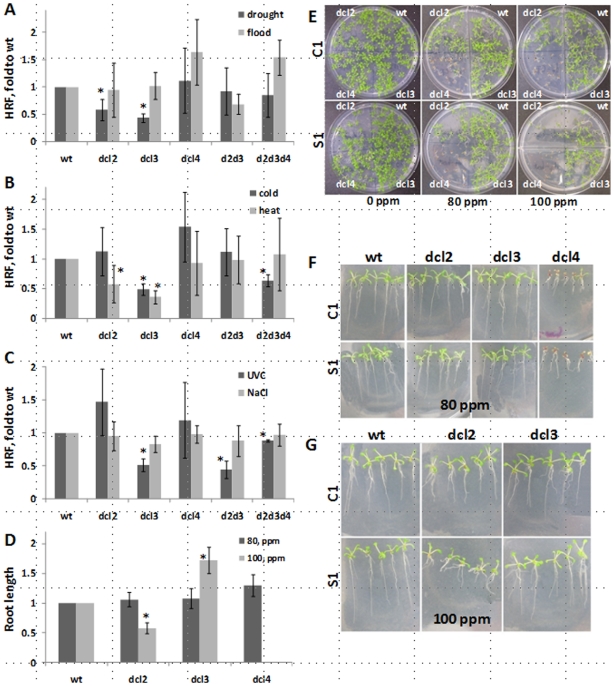
Changes in HRF and stress tolerance in DCL mutants. **A.** HRF in the C1 and S1 progeny of the wt, *dcl2*, *dcl3*, *dcl4*, *dcl2 dcl3* and *dcl2 dcl3 dcl4* plants exposed to drought and flood stress. The ‘Y’ axis shows HRF (the average of 3 experiments and s.e.m.) as fold of S1 to C1, standardized to wt. Asterisks show significant differences in mutants as compared to wt (*p*<0.05; a single-factor ANOVA). **B.** HRF in the C1 and S1 progeny of the wt, *dcl2*, *dcl3*, *dcl4*, *dcl2 dcl3* and *dcl2 dcl3 dcl4* plants exposed to heat and cold stress. The ‘Y’ axis shows HRF (the average of 3 experiments and s.e.m.) as fold of S1 to C1, standardized to wt. **C.** HRF in the C1 and S1 progeny of the wt, *dcl2*, *dcl3*, *dcl4*, *dcl2 dcl3* and *dcl2 dcl3 dcl4* plants exposed to UVC and NaCl stress. The ‘Y’ axis shows HRF (the average of 3 experiments and s.e.m.) as fold of S1 to C1, standardized to wt. **D.** Root length of the C1 and S1 progeny of the heat-treated wt, *dcl2*, *dcl3* and *dcl4* plants germinated and grown in the presence of 80 and 100 ppm MMS. Root length was measured in 20 plantlets from each experimental group. The ‘Y’ axis shows a ratio of S1 to C1, standardized to wt. **E.** Representative Petri plates of the C1 (upper panel) and S1 (lower panel) progeny of the heat-treated wt (top right corner of each plate), *dcl2* (top left corner), *dcl3* (bottom right corner) and *dcl4* (bottom left corner) plants germinated and grown in the presence of 80 and 100 ppm MMS. **F.** Representative plants of the C1 and S1 progeny of the heat-treated wt, *dcl2*, *dcl3* and *dcl4* plants germinated and grown in the presence of 80 ppm MMS. **G.** Representative plants of the C1 and S1 progeny of the heat-treated wt, *dcl2* and *dcl3* plants germinated and grown in the presence of 100 ppm MMS.

The analysis of HRF showed that *dcl2*, *dcl3* as well as *dcl2 dcl3* and *dcl2 dcl3 dcl4* mutants are partially impaired in a stress-induced increase in response to drought, heat, cold and UVC. The double *dcl2 dcl3* mutant was deficient in a stress-induced increase in HRF in response to UVC; whereas, the triple *dcl2 dcl3 dcl4* mutant was deficient in response to UVC and cold. Interestingly, the *dcl3* mutant was also impaired in recombination increase in response to UVC and cold, whereas the *dcl2* mutant was not (Figure 7A–C). Exposure of C1 and S1 progenies of heat-treated wild type plants and *dcl2*, *dcl3* and *dcl4* mutants to 80 and 100 ppm MMS showed that the S1 progeny of *dcl2* plants is less tolerant to stress, whereas *dcl3* is more tolerant as compared to the C1 progeny (Figure 7D–G). The C1 and S1 progenies of *dcl4* plants were extremely sensitive to MMS, although no difference between the C1 and S1 plants was observed (Figure 7D–G). Thus, although DCL2 and DCL3 are required, the two dicers appear to play different roles in response to abiotic stress and the adaption process.

**Figure 8:**
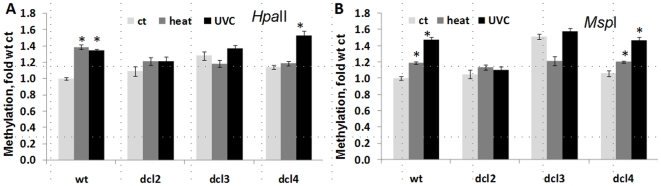
Changes in methylation in DCL mutants. The progeny of the control (C1) plants and the progeny of plants exposed to heat and UVC (S1) belonging to wild type, *dcl2*, *dcl3* and *dcl4* groups were used for the analysis. Genomic DNA was prepared from 20 three-week-old plants per each experimental group, and methylation was measured via the cytosine extension assay using digestion with *Hpa*II and *Msp*I as described before [14]. The data are shown as fold of methylation relative to the wild type C1 (ct) plants as calculated from 3 independent repeats. Significant differences between the C1 and S1 plants in each group are shown by asterisks (p<0.05). A. Data for *Hpa*II. B. Data for *Msp*I.

Methylation analysis in the progeny of plants exposed to heat and UVC showed that wild-type plants exhibit genome-wide hypermethylation. The progeny of *dcl4* plants exposed to UVC also exhibited genome hypermethylation. In contrast, the progeny of stressed *dcl2* and *dcl3* plants did not show significant changes in methylation as compared to the progeny of control plants (Figure 8A,B). To summarize, these results show that *dcl2* and *dcl3* are partially impaired in the establishment of transgenerational changes in HRF and DNA methylation in the progeny of heat-stressed plants.

## Discussion

Acclimation and adaptation to stress are well known types of transgenerational adaptive plasticity [26]. Examples include tolerance to several stresses in timberline plants associated with adaptation to UV-B radiation [7]; increased tolerance to cold in progeny of *Arabidopsis* plants grown at low temperatures [5]; and enhanced performance of progeny grown in the light environment of parents in a parental light environment [26]. We found that progeny of *Arabidopsis* plants exposed to salt, temperature, water and UVC stresses exhibit increased HRF, increased tolerance to stress, and increased DNA methylation.

The heritability of these transgenerational effects in successive generations is still an issue. Although we were able to confirm earlier studies [3] reporting that several stresses, including UVC, induced changes in HRF that persisted in the progeny, we could not prove that these effects persisted in successive generations in the absence of stress. In a recent study, Pecinka et al. (2009) reported that transgenerational effects of stress on HFR were stochastic, i.e., highly variable and dependent on the nature of stress [15]. Four of 10 stress conditions they tested appeared to be effective: a genotoxic agent bleomycin and chemical zebularine which blocks cytosine methylation induced a persistent increase in HFR. Paraquat which induces oxidative stress increased HRF that did not persist; and mannitol which induces osmotic stress decreased HRF in the progeny; these results are consistent with the effect of drought that we observed. Factors that might account for the discrepancies include transgenic lines used, plant age and growth conditions as well as the exact nature of stress protocols. In conclusion, various stress factors can induce transgenerational changes in HRF; however, these changes do not represent a consistent general response to stress, and moreover, they are not necessarily inherited in the absence of stress. Further, since these measurements depend on the use of transgenes as reporters, the biological significance of observations is still unclear.

Changes in DNA methylation have been proposed to be responsible for adaptation to stress by *Arabidopsis thaliana* plants and the pine tree population naturally grown in the vicinity of Chernobyl [12], [13]. Common iceplants (*Mesembryanthemum crystallinum*) exposed to stress undergo changes in satellite DNA methylation, which results in a switch from C3-type to C4-type carbon dioxide assimilation [27]. A direct correlation between the frequency of rearrangements at various disease-resistant gene-like loci and the level of methylation at these loci in response to stress resulting from virus infection was observed before [14]. In the present study we found that transgenerational effects of stress on HRF and stress tolerance were associated with changes in DNA methylation. Together with the effects of 5azaC on stress tolerance, this is consistent with the hypothesis that alterations in DNA methylation are required for transgenerational effects that we observed. The exact relationship between DNA methylation and stress is still unclear. While the genome of S1 plants was hypermethylated at the global level, many loci nonetheless exhibited hypomethylation. Moreover, we found no clear correlation between changes in HRF at the transgene locus and methylation of the locus.

**Figure 9:**
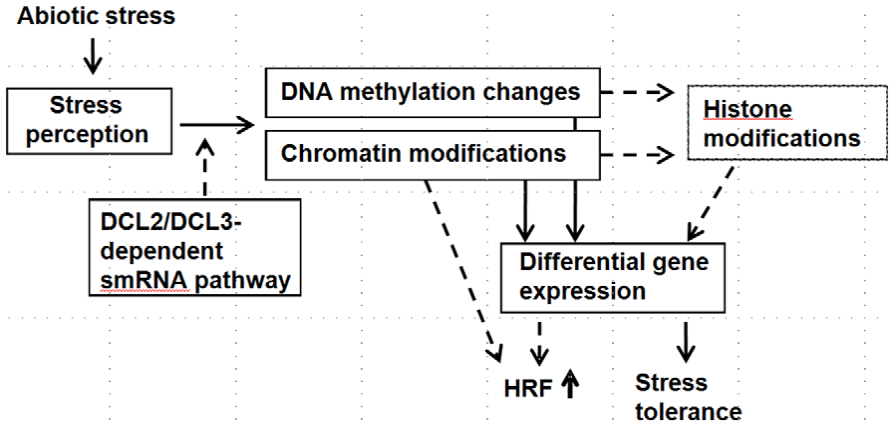
Potential mechanism of transgenerational changes in the progeny of stressed plants. We hypothesize that exposure to stress triggers changes in plants that lead to transgenerational changes in methylation and possibly in chromatin modifications. This process is apparently dependent on the function of small RNAs. Chromatin modifications may be sufficient to trigger an increase in recombination frequency. Differential genome methylation and changes in chromatin structure could lead to differential gene expression that could also be a cause of the increase in stress tolerance and recombination frequency. Chromatin modifications could involve histone modifications, resulting in a differential pattern of hetero-/euchromatin and thus in changes in HRF and stress tolerance.

Interestingly, several genes known to be involved in HFR or chromatin modifications showed altered methylation in S1_25 and S1_75 plants relative to controls (Table S2, S3; Figure 9). For example, the promoter region of Msh2 involved in mismatch repair and UVH3 involved in UV-damaged DNA repair exhibited a 50% decrease in methylation associated with stress. Similarly, 5 different genes encoding proteins that are involved in histone modification, namely, SUVH2, SUVH5, SUVH6, FLD and UBP26, showed a dramatic increase in methylation associated with stress. SUVH2, SUVH5 and SUVH6 are histone methyltransferases involved in heterochromatic gene silencing [28]. *SUVH5* together with *SUVH4 (KRYPTONITE)* control transposon movement, whereas *SUVH6* together with *SUVH4* control transcribed inverted repeats [29]. Flowering locus D (FLD) encodes a protein containing a histone deacetylation domain. Deficiency in FLD results in hyperacetylation of FLC chromatin, up-regulation of FLC expression, and extremely delayed flowering [30]. *UBP26* encodes an enzyme that removes ubiquitin modifications of histone H2B, facilitates DNA methylation and heterochromatin formation, and is important for endosperm and flowering [31].

Our results provide evidence that transgenerational effects of salt stress on HRF and stress tolerance depend on DCL2 and/or DCL3. Small RNA biogenesis in *Arabidopsis thaliana* depends on several proteins including DCLs. In particular, biogenesis of miRNA requires DCL1, whereas siRNA biogenesis depends on DCL2, DCL3, and DCL4.

Stress is known to induce the differential expression of various small regulatory RNAs [32], [33]. Micro-RNAs (miRNAs) seem to be the predominant class of molecules that are induced by abiotic stress such as cold, drought, salt and UV, with many of them being commonly regulated [32], [33]. The involvement of siRNAs in abiotic stress response is somewhat less established, the salt-regulated nat-siRNA P5CDH and SRO5 pair being the most well-known example [34]. The involvement of siRNA metabolism in the establishment of a new methylation pattern and possibly stress tolerance has been suggested before [35], [36]. Recent work by Agorio and Vera (2007) showed the role of AGO4 in the process of resistance of *Arabidopsis* to *Pseudomonas syringae*; these scientists found that *ago4* was sensitive to bacterial infection [37]. It is an interesting fact that *dcl3* and *rdr2* mutants, which are supposedly impaired in the same pathway of siRNA biogenesis, remained tolerant to bacterial infection, thus suggesting the complex process of stress tolerance this pathway is involved in.

Each of the DCL enzymes generates predominantly a particular class of small RNAs. Whereas DCL1 is required for miRNA biogenesis [38], DCL2 is apparently needed for the generation of viral siRNAs [39]. DCL3 is involved in processing of endogenous repeats and in the formation of heterochromatic siRNAs [39], whereas DCL4 is required for ta-siRNA biogenesis [40]. DCL3-dependent processing of endogenous repeats and the formation of heterochromatic siRNAs can be considered as one of the mechanisms capable of directing RNA-dependent DNA methylation [41].

The involvement of DCL2 and DCL3 in passing on the memory of stress to progeny may occur at different levels, the main one possibly being the establishment of a differential methylation pattern via the activity of small RNAs. The reason why the picture of the involvement of DCLs in transgenerational response did not become more pronounced can be explained by the substantial functional redundancy of DCLs, suggesting their compensating functions [42], [43].

In conclusion, we have shown that the progeny of stressed plants exhibit changes in recombination frequency, genome methylation and stress tolerance. Admittedly, we have only documented these changes in HRF within the transgene reporter thus far. No mechanistic link between DNA methylation and the level of homologous recombination at the loci we have studied can be established here. However, we provide the first experimental evidence that the establishment of stress acclimation and stress adaptation correlates with changes in genome methylation and potentially depends on small RNA pathways requiring DCL2 and DCL3. It remains to be determined whether stress induces the expression of smRNAs targeting specific sequences within the plant genome for methylation or repressive histone modifications. Experiments involving various mutants impaired in the establishment/maintenance of methylation patterns, such as *drm2*, *ddm1*, *met1*, *cmt3* as well as mutants impaired in biogenesis of miRNAs/siRNAs, will provide further insight into this interesting phenomenon.

## Materials and Methods

### *Arabidopsis* Plant Lines Used for the Experiment

Two transgenic *Arabidopsis thaliana* recombination reporter lines, line 11 (ecotype C24) and line 15d8 (ecotype Col-0) were used for the experiments [16], [17]. DCL mutants *dcl2-5* [in Col-0], *dcl3-1* [in Col-0], *dcl4-2* [in Col-0], *dcl2 dcl3*, *dcl2 dcl3 dcl4* (ref. [42]) were crossed with the recombination reporter line 15d8 in the Col-0 background. Plants homozygous for the recombination reporter transgene and respective DCL mutants were selected and used for further analysis.

### Experimental Set-Up

*Arabidopsis* plants (G0) were propagated to the next generation (G1) under normal growth conditions (C1) or in the presence of stress (S1 for ‘stressed, generation 1’) (Figure 1A). Next, the S1 plants were propagated to G2 in the presence of stress (S2) or under normal conditions (S1C1). The C1 plants were propagated to G2 under normal conditions (C2).

Unless indicated otherwise, the plants were grown in soil at 22°C under 12 h day/12 h night conditions and illumination at 100 µM m^−2^ sec^−1^. For analyzing the effect of NaCl stress, the *Arabidopsis* plants from line 11 were germinated and grown on sterile MS media supplemented with either 25 or 75 mM NaCl. Three weeks later, the plants were transferred into soil. To analyze the effect of exposure to other stresses, the *Arabidopsis* plants from line 15d8 were used. To investigate responses to heat, cold, flood and drought stress, *Arabidopsis* plants were germinated and grown in soil. Flood stress was created by watering plants every day, making sure that the pots were standing in water all the time. Drought stress was created by stopping watering between 7–30 dpg. For analyzing the impact of heat stress, the plants were germinated in soil and exposed to 37°C for 3h/day during the day for one week starting at 7 dpg. The effect of cold stress was investigated on the plants that were germinated in soil and at 7 dpg were exposed to 4°C for 12 hours during the night for one week. To analyze the effect of UVC stress, plantlets were exposed to 5,000 erg UVC every day for 4 days starting at 7 dpg. C1 and S1 plants from DCL mutants exposed to heat, cold, drought, flood, salt and UVC were generated in the same manner as wild-type plants. In each case, 20 plants were used to produce the next generation. Seeds from these plants were pooled together and used for further experiments.

Tolerance to methyl methanesulfonate (MMS) stress was measured by germinating *dcl* mutants on MS medium supplemented with 0, 80, 100 and 120 ppm MMS. Approximately 50 seeds per each concentration of MMS were used. Root length was measured in 20 plantlets per each experimental group (Figure 7D), and pictures were taken at 18 days after exposure (Figure 7E–G).

### Analysis of HRF

HRF in *Arabidopsis* plants carrying a GUS transgene was analyzed after histochemical staining (Figure 1B,C) as described [44]. HRF in *Arabidopsis* plants of line #15d8 carrying the luciferase transgene was analyzed by scoring bright sectors on a dark background in a luciferase CCD camera after spraying with luciferin [17]. In line #11, we used 200 three-week-old plantlets per each experimental group, whereas in line #15d8, 50 plantlets per group were used. HRF was calculated by relating the number of events to the total number of plants scored. Each experiment was repeated at least 3 times.

### Analysis of Global Genome Methylation

Genomic DNA was prepared from 20 plantlets using trizol reagent as published before [14]. DNA was digested for 48 h with a 10-fold excess of either *Hpa*II or *Msp*I endonuclease according to the manufacturer's protocol (New England Biolabs, Beverly, MA). An additional DNA aliquot was incubated without any restriction enzyme as a background control. A single-nucleotide extension reaction was performed in 2 µg of DNA using the cytosine extension assay described previously [14]. The data obtained from 3 independent experimental groups with 2 measurements per each group are expressed as a percentage of dpm/µg of DNA relative to background controls.

### Immunoprecipitation and Microarray Analysis of Methylated DNA

Genome-wide analysis of DNA methylation was performed as described [45] (Figure S1). Genomic DNA prepared from leaves of 20 three-week-old A. thaliana plants (C1, S1_25 and S1_75) was sheared by sonication to 500- to 1,500-bp fragments, and methylated DNA was immunoprecipitated as described [45]. The entire immunoprecipitation reaction and 500 ng of control DNA were amplified using the T7 RNA polymerase linear amplification protocol as described [45]. Immunoprecipitated DNA was labelled with Cy5, and control DNA–with Cy3 fluorescent dyes. The labelled samples were hybridized to Whole Genome Tilling Array 2 (Catalog number C4348001-02-01, Nimblgen). Tilling Array 2 contains positions 9,687,916 to 19,704,755 of chromosome 2, the entire sequence of chromosome 3, and positions 1,001 to 6,133,069 of chromosome 4. For MeDIP analysis, array intensities are represented as log_2_ signal ratios of immunoprecipitated DNA to input DNA. Data normalization for Cy3- and Cy5-labeled samples was performed via linear regression of log(Cy3) versus log(Cy5) as well as via mean/median correction, followed by correction using the intensity of random data sets. The data are shown as the log_2_ ratio of intensities of immunoprecipitated to input DNA.

Further analysis of methylation was done by using the 5 kb sequence 5′ from the transcribed region defined as a promoter region and the transcribed sequence itself. We compared differences in methylation between S1_25 and C1 as well as S1_75 and C1 plants. Regions were scored as hypermethylated if S1_25 or S1_75 plants had a higher level of methylation (50% or 80%) as compared to C1 plants and they were scored as hypomethylated if they had a lower level of methylation (50% and 80%). The regions of the promoter and transcribed sequences were considered for analysis if at least 5 probes (each of 90 bp) were scored positive for changes between enriched and input DNA.

### Bisulfite Sequencing of the 35S Promoter

Bisulfite converts unmethylated cytosins to uracils, whereas methylated cytosins stay unconverted. PCR amplification then converts uracils to thymines. Bisulfite conversion of DNA was done using the EZ DNA Methylation-Gold Kit (Zymo Research Corp.) according to the manufacturer's protocol. Amplification of 181 bp DNA fragments was carried out using the AmpliTaq Gold DNA Polymerase (Applied Biosystems). PCR conditions: 1) 95°C for 2 minutes; 2) 95°C for 30 sec, 60°C for 30 sec, 72°C for 30 sec, repeated 35 times; 3) 72°C for 2 minutes. PCR fragments were analyzed by electrophoresis in a 1% agarose gel (1x TAE buffer) and cloned into pJET1.2/blunt vector using the CloneJET PCR Cloning Kit (Fermentas). Recombinant clones were screened by PCR. 16 positive clones per each group (C1 or S1_25) were selected randomly for sequencing. Analysis of DNA sequences was done using a BiQ Analyzer software tool [46]. Primer design was done using the MethPrimer software tool [47].

Primers: AG276 (Froward) (201–227 bp in 35S promoter), AG277 (Reverse) (358–381 bp in 35S promoter). Sodium bisulfite-treated DNA from the C1 and S1_25 progeny of salt-treated plants was PCR-amplified using the 35S promoter specific primers: forward 5′ TGAGATTTTTTAATAAAGGGTAATATT 3′, reverse 5′ TGAGATTTTTTAATAAAGGGTAATATT 3′ (Figure S2). Sequences with the conversion rate over 70% were used for comparison.

### Microchip Analysis of Gene Expression

Global transcriptome of S1_25 and C1 plants was analyzed by preparing total RNA from three-week old plants. RNA labeling and hybridization to the Affymetrix ATH1 array and posthybridization scanning and data pre-processing was conducted by the Genome Quebec Core Facility. Three independent sets of RNA samples were analyzed for S1_25 and three for C1 plants. The datasets were analyzed using “FlexArray” software developed by M. Blazejczyk and associates (Genome Quebec, Montreal). In brief, raw data thus were adjusted for background and normalized using the Robust Microchip Average (RMA) method [48]. To identify set of differentially regulated genes we performed three independent analyses, equal-variance t-statistic, Significance Analysis of Microarrays (SAM) and algorithm EB (empirical Bayes) Wright & Simon.

The cut-offs used were: a 2-fold change and *p*<0.05, and a 3-fold change and *p*<0.01.

### Pretreatment with 5-AzaC and Analysis of Stress Response

Seeds of C1, S1_25 and S1_75 plants were germinated on half-MS medium. Five experimental groups were formed. Each experimental group contained 20 plantlets. Plants of a control group remained in this medium for the entire length of the experiment. At 3 dpg, plants belonging to a ‘transfer’ group were transferred to similar half-MS medium and served as a ‘transfer’ control. At 3 dpg, plants of the ‘5-AzaC’ group were transferred to 50 µM 5-azaC. At 3 dpg, plants of the ‘5-AzaC/NaCl’ group were transferred to 50 µM 5-azaC, and at 8 dpg, they were transferred from media containing 5-azaC to media containing 100 mM NaCl. At 8 dpg, plants of the ‘NaCl’ group were transferred to 100 mM NaCl. Plants from all experimental groups were harvested at 19 dpg. Genomic DNA was prepared and digested with either *Hpa*II or *Msp*I, and global genome methylation was analyzed. The experiment was repeated three times.

For analysis of stress tolerance, C1, S1_25 and S1_75 plants pre-treated with 5-azaC, and control plants were moved to media containing different amount of NaCl, 0, 100, 150 and 200 mM (Figure 4B). Roots were measured in 20 plantlets per each experimental group, and the picture was taken; 3 representative plantlets from each experimental group were placed on media, and the pictures of plantlets and roots were taken. The experiment was repeated three times.

### Statistical Treatment of the Data

Statistical analyses were performed using MS Excel software and Microcal Origin 6.0. Standard errors or standard deviations were calculated. Statistical significance between the means was compared using either Student's t-test or single factor ANOVA.

## Supporting Information

Figure S1 Progeny of salt-stressed plants exhibit higher tolerance to MMS. C1, S1_25 and S1_75 plants were used for the analysis of tolerance to MMS. Thirty to forty plants per each experimental group were geminated on normal media or media supplemented with 100, 120 or 130 ppm MMS. The picture was taken after two weeks of exposure.
(3.35 MB TIF)

Figure S2 Bisulfite sequencing of the 35S promoter in C1 and S1_25 plants. Bisulfite sequencing was performed after bisulfite conversion and amplification using the 35S-specific primers. A. Shows the sequence of the 35S promoter. Primers are shown in red. B. The first line labeled as “Original GenSeq” shows the original genomic sequence, whereas the second line labeled as “Fully converted GenSeq” shows the sequence of fully bisulfite-converted DNA. “C1” shows seven sequences in C1 plants, whereas “S1_25” shows seven sequences in S1_25 plants. CG nucleotides are shown in bold, whereas CNG are underlined. Unconverted cytosines at CG sites are in orange, whereas converted ones are in violet. Unconverted cytosines at non CG sites are shown in turquoise. Regions without any changes in methylation are excluded (“+31 bp” and two “+10 bp” regions).
(2.81 MB TIF)

Figure S3 Analysis of global genome methylation using MeDIP. Genomic DNA prepared from leaves of three-week-old *A. thaliana* plants (C1, S1_25 and S1_75) was sheared by sonication to 500- to 1,500-bp fragments, and methylated DNA was immunoprecipitated as described (Zilberman et al., 2006). The entire immunoprecipitation reaction and 500 ng of control DNA were amplified using the T7 RNA polymerase linear amplification protocol as described (Zilberman et al., 2006). Immunoprecipitated DNA was labelled with Cy5, and control DNA - with Cy3 fluorescent dyes. The labelled samples were hybridized to Whole Genome Tilling Array 2 (Catalog number C4348001-02-01, Nimblgen). Tilling Array 2 contains positions 9,687,916 to 19,704,755 of chromosome 2, the entire sequence of chromosome 3, and positions 1,001 to 6,133,069 of chromosome 4. For MeDIP analysis, array intensities are represented as log2 signal ratios of immunoprecipitated DNA to input DNA. Data normalization for Cy3- and Cy5-labeled samples was performed via linear regression of log(Cy3) versus log(Cy5) as well as via mean/median correction, followed by correction using the intensity of random data sets. The data are shown as the log2 ratio of intensities of immunoprecipitated to input DNA. A–C show all data for ch.2, ch.3 and ch.4, whereas D–G show the ratio for specific areas of chromosomes 2, 3 and 4. D–F show hypomethylation of DNA of S1_25 and S1_75, whereas G shows hypermethylation.
(2.55 MB TIF)

Figure S4 Semi-quantitative RT-PCR confirms the validity of microchip data. For SQ RT-PCR analysis we chose the following genes: At1g43160, At1g61560, At2g27690, At3g50970, At4g25470, At5g61600. For the analysis, C1 and S1_25 plants were grown for three weeks on soil. PCR prepared from three biological repeats per each group were used to produce cDNA. A. Figure shows the average (with SE) arbitrary units of intensity as measured from three independent SQ RT-PCRs. The data were standardized to tubulin. Asterisks show significant differences (p<0.001) between S1_25 and C1 plants. The picture below shows the representative image of SQ RT-PCR. The insert shows the control amplification of tubulin. B. Table shows the fold difference in the expression of 6 above mentioned genes as calculated between S1_25 and C1 plants. The data are shown for microchip analysis (Chip) and for SQ RT-PCR analysis (PCR).
(1.90 MB TIF)

Figure S5 Pre-treatment of the progeny of salt-stressed plants with 5-azaC decreases their tolerance to NaCl. C1, S1_25 and S1_75 were germinated on liquid MS supplemented with or without 5-azaC and at the age of one week were moved to 0, 100, 120 and 130 ppm MMS. A - A representative picture of one of 3 plates. B - Root length (the average from 3 independent plates, 5 plants per each plate, with s.e.m.). Note higher tolerance of the S1 plants grown without 5-azaC and equal tolerance of the S1 plants grown with 5-azaC, as compared to the C1 plants. Asterisks show significant differences between the S1_25 and S_75 and C1 groups of plants exposed to 120 ppm MMS and not exposed to 5-azaC (a single-factor ANOVA, p<0.05, for both). A two-factor ANOVA, with one being levels of MMS exposure (100, 120 and 130 ppm) and treatments of parental lines (C, S1_25, S1_75), showed significant changes for both MMS treatment and the parental line (p<0.001 and p<0.05, respectively) in plants that were not exposed to 5-azaC. In contrast, the two-factor ANOVA performed for plants that were pretreated with 5-azaC showed significant differences for MMS treatment (p<0.05) but not for the parental lines (p = 0.93).
(2.26 MB TIF)

Table S1 Data are shown as the average recombination frequency in G0, S1, S2 and S1C1 plants stemming from exposure to drought, flood, heat, cold and UVC or propagated at non-induced conditions (control line 11 and control line 15d8).
(0.04 MB DOC)

Table S2 List of genes with over 80% hypermethylation either at promoter or transcribed regions of the S1_25 and S1_75 plants.
(0.08 MB DOC)

Table S3 List of genes with over 80% hypomethylation either at promoter or transcribed regions of the S1_25 and S1_75 plants.
(0.05 MB DOC)

Table S4 “Fold” shows fold difference between S1_25 and C1 plants.
(0.08 MB DOC)
