# Correction: Transgenerational Adaptation of *Arabidopsis* to Stress Requires DNA Methylation and the Function of Dicer-Like Proteins

**DOI:** 10.1371/annotation/726f31b5-99c4-44e9-9cd6-b8d66b3f6038

**Published:** 2010-04-29

**Authors:** Alex Boyko, Todd Blevins, Youli Yao, Andrey Golubov, Andriy Bilichak, Yaroslav Ilnytskyy, Jens Hollunder, Frederick Meins, Igor Kovalchuk

The seventh author's name was spelled incorrectly. The correct name is: Jens Hollunder.

