# Supplementary figures and images for: Transgenerational Adaptation of Arabidopsis to Stress Requires DNA Methylation and the Function of Dicer-Like Proteins
Source: PLoS One. 2010 Mar 3;5(3):e9514. doi: 10.1371/journal.pone.0009514 (PMC2831073; doi:10.1371/journal.pone.0009514)

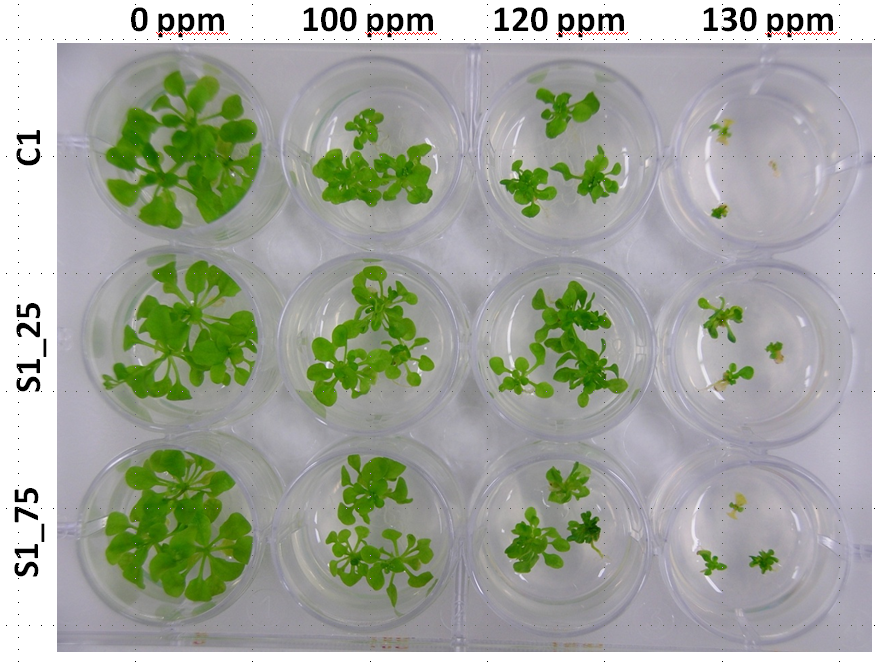

Supplement: Figure S1 — Progeny of salt-stressed plants exhibit higher tolerance to MMS. C1, S1_25 and S1_75 plants were used for the analysis of tolerance to MMS. Thirty to forty plants per each experimental group were geminated on normal media or media supplemented with 100, 120 or 130 ppm MMS. The picture was taken after two weeks of exposure. (3.35 MB TIF) [file pone.0009514.s001.tif]

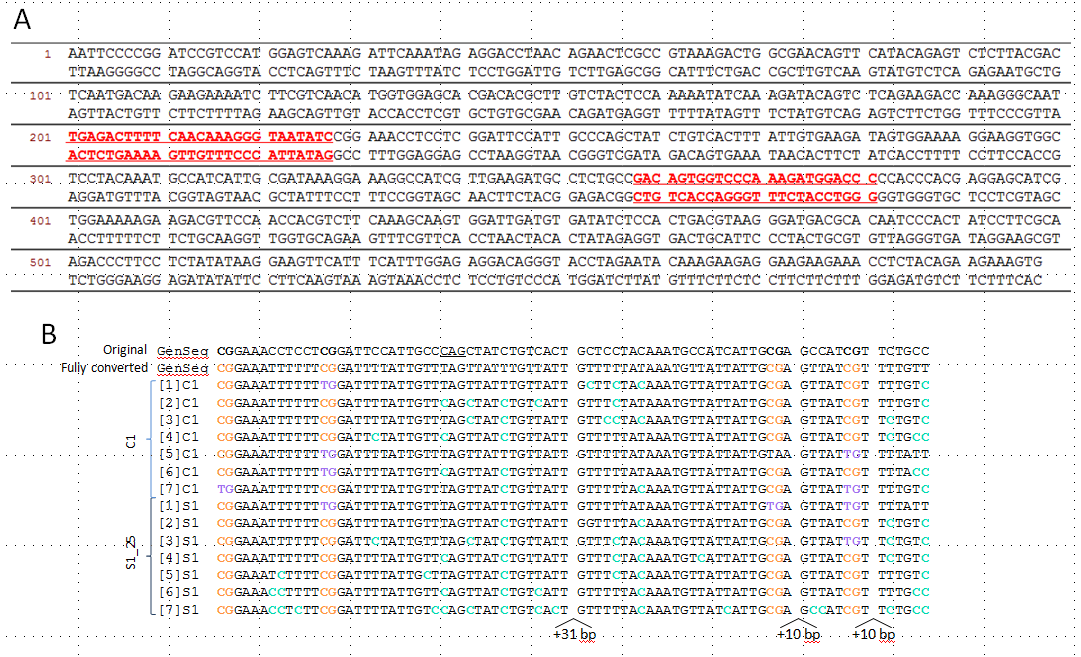

Supplement: Figure S2 — Bisulfite sequencing of the 35S promoter in C1 and S1_25 plants. Bisulfite sequencing was performed after bisulfite conversion and amplification using the 35S-specific primers. A. Shows the sequence of the 35S promoter. Primers are shown in red. B. The first line labeled as “Original GenSeq” shows the original genomic sequence, whereas the second line labeled as “Fully converted GenSeq” shows the sequence of fully bisulfite-converted DNA. “C1” shows seven sequences in C1 plants, whereas “S1_25” shows seven sequences in S1_25 plants. CG nucleotides are shown in bold, whereas CNG are underlined. Unconverted cytosines at CG sites are in orange, whereas converted ones are in violet. Unconverted cytosines at non CG sites are shown in turquoise. Regions without any changes in methylation are excluded (“+31 bp” and two “+10 bp” regions). (2.81 MB TIF) [file pone.0009514.s002.tif]

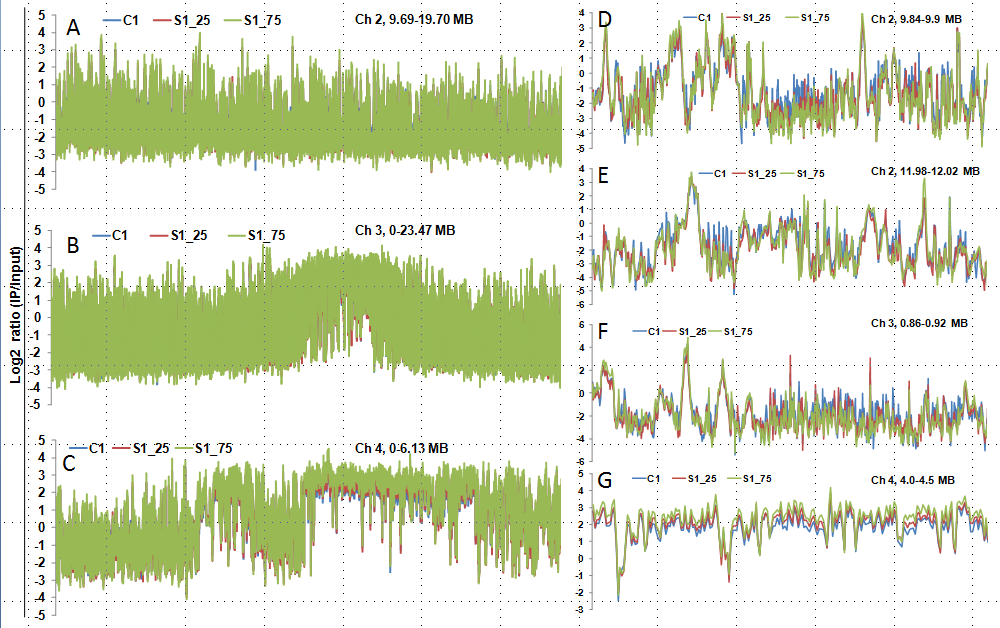

Supplement: Figure S3 — Analysis of global genome methylation using MeDIP. Genomic DNA prepared from leaves of three-week-old A. thaliana plants (C1, S1_25 and S1_75) was sheared by sonication to 500- to 1,500-bp fragments, and methylated DNA was immunoprecipitated as described (Zilberman et al., 2006). The entire immunoprecipitation reaction and 500 ng of control DNA were amplified using the T7 RNA polymerase linear amplification protocol as described (Zilberman et al., 2006). Immunoprecipitated DNA was labelled with Cy5, and control DNA - with Cy3 fluorescent dyes. The labelled samples were hybridized to Whole Genome Tilling Array 2 (Catalog number C4348001-02-01, Nimblgen). Tilling Array 2 contains positions 9,687,916 to 19,704,755 of chromosome 2, the entire sequence of chromosome 3, and positions 1,001 to 6,133,069 of chromosome 4. For MeDIP analysis, array intensities are represented as log2 signal ratios of immunoprecipitated DNA to input DNA. Data normalization for Cy3- and Cy5-labeled samples was performed via linear regression of log(Cy3) versus log(Cy5) as well as via mean/median correction, followed by correction using the intensity of random data sets. The data are shown as the log2 ratio of intensities of immunoprecipitated to input DNA. A–C show all data for ch.2, ch.3 and ch.4, whereas D–G show the ratio for specific areas of chromosomes 2, 3 and 4. D–F show hypomethylation of DNA of S1_25 and S1_75, whereas G shows hypermethylation. (2.55 MB TIF) [file pone.0009514.s003.tif]

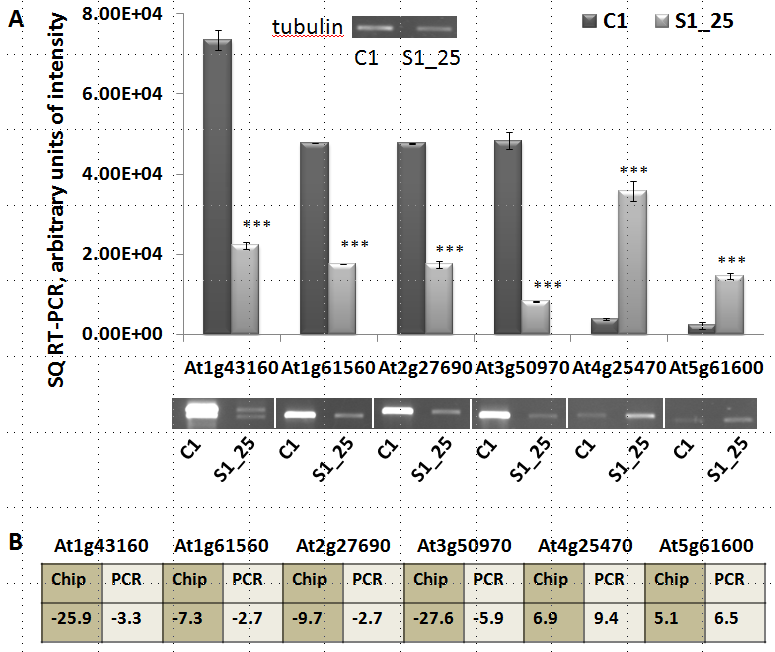

Supplement: Figure S4 — Semi-quantitative RT-PCR confirms the validity of microchip data. For SQ RT-PCR analysis we chose the following genes: At1g43160, At1g61560, At2g27690, At3g50970, At4g25470, At5g61600. For the analysis, C1 and S1_25 plants were grown for three weeks on soil. PCR prepared from three biological repeats per each group were used to produce cDNA. A. Figure shows the average (with SE) arbitrary units of intensity as measured from three independent SQ RT-PCRs. The data were standardized to tubulin. Asterisks show significant differences (p<0.001) between S1_25 and C1 plants. The picture below shows the representative image of SQ RT-PCR. The insert shows the control amplification of tubulin. B. Table shows the fold difference in the expression of 6 above mentioned genes as calculated between S1_25 and C1 plants. The data are shown for microchip analysis (Chip) and for SQ RT-PCR analysis (PCR). (1.90 MB TIF) [file pone.0009514.s004.tif]

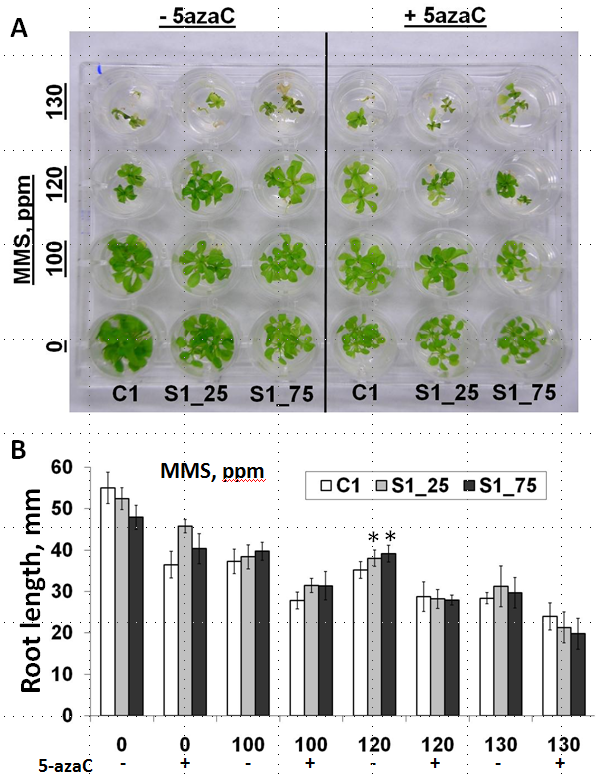

Supplement: Figure S5 — Pre-treatment of the progeny of salt-stressed plants with 5-azaC decreases their tolerance to NaCl. C1, S1_25 and S1_75 were germinated on liquid MS supplemented with or without 5-azaC and at the age of one week were moved to 0, 100, 120 and 130 ppm MMS. A - A representative picture of one of 3 plates. B - Root length (the average from 3 independent plates, 5 plants per each plate, with s.e.m.). Note higher tolerance of the S1 plants grown without 5-azaC and equal tolerance of the S1 plants grown with 5-azaC, as compared to the C1 plants. Asterisks show significant differences between the S1_25 and S_75 and C1 groups of plants exposed to 120 ppm MMS and not exposed to 5-azaC (a single-factor ANOVA, p<0.05, for both). A two-factor ANOVA, with one being levels of MMS exposure (100, 120 and 130 ppm) and treatments of parental lines (C, S1_25, S1_75), showed significant changes for both MMS treatment and the parental line (p<0.001 and p<0.05, respectively) in plants that were not exposed to 5-azaC. In contrast, the two-factor ANOVA performed for plants that were pretreated with 5-azaC showed significant differences for MMS treatment (p<0.05) but not for the parental lines (p = 0.93). (2.26 MB TIF) [file pone.0009514.s005.tif]
