# Supplementary material for: Transgenerational Adaptation of Arabidopsis to Stress Requires DNA Methylation and the Function of Dicer-Like Proteins
Source: PLoS One. 2010 Mar 3;5(3):e9514. doi: 10.1371/journal.pone.0009514 (PMC2831073; doi:10.1371/journal.pone.0009514)
Supplement: Table S1 — Data are shown as the average recombination frequency in G0, S1, S2 and S1C1 plants stemming from exposure to drought, flood, heat, cold and UVC or propagated at non-induced conditions (control line 11 and control line 15d8). (0.04 MB DOC) [file pone.0009514.s006.doc]

|  | G0 | S1 | S2 | S1C1 |
| --- | --- | --- | --- | --- |
| Control line 11 | 0.36±0.022 | 0.35±0.092 | 0.29±0.033 | 0.29±0.033 |
| 25 mM NaCl | 0.84±0.19 | 0.76±0.091 | 0.67±0.049 | 0.43±0.041 |
| 75 mM NaCl | 1.26±0.22 | 0.49±0.074 | 0.46±0.093 | 0.33±0.046 |
|  |  |  |  |  |
| Control line 15d8 | 3.72±0.31 | 3.52±0.44 | 3.69±0.28 | 3.69±0.19 |
| Drought | 2.20±0.12 | 2.64±0.21 | 2.66±0.11 | 3.76±0.52 |
| Flood | 8.31±2.1 | 6.69±0.69 | 6.64±0.39 | 4.80±0.25 |
| Heat | 7.89±0.9 | 6.12±0.74 | 9.15±1.12 | 5.20±0.73 |
| Cold | 5.78±0.4 | 4.22±0.88 | 6.13±0.55 | 4.21±0.19 |
| UVC | 18.53±2.8 | 9.49±1.33 | 12.58±2.2 | 5.72±0.68 |

**Table S1. HRF in line 11 and line 15d8 exposed to stress**
