# Supplementary material for: Transgenerational Adaptation of Arabidopsis to Stress Requires DNA Methylation and the Function of Dicer-Like Proteins
Source: PLoS One. 2010 Mar 3;5(3):e9514. doi: 10.1371/journal.pone.0009514 (PMC2831073; doi:10.1371/journal.pone.0009514)
Supplement: Table S2 — List of genes with over 80% hypermethylation either at promoter or transcribed regions of the S1_25 and S1_75 plants. (0.08 MB DOC) [file pone.0009514.s007.doc]

**Table S2. List of genes with over 80% hypermethylation either at promoter or transcribed regions of the S1_25 and S1_75 plants**

|  | **Locus ID** | **Gene Annotation** | **Possible role** |
| --- | --- | --- | --- |
| **25_**  **promoter** | [AT3G29205](http://www.arabidopsis.org/servlets/TairObject?name=AT3G29205&type=locus) | non-LTR retrotransposon family (LINE) | Transposon |
|  | [AT3G42251](http://www.arabidopsis.org/servlets/TairObject?name=AT3G42251&type=locus) | transposable element gene | Transposon |
|  | [AT4G08980](http://www.arabidopsis.org/servlets/TairObject?name=AT4G08980&type=locus) | F-box family protein (FBW2) | Signalling/Cell cycle |
|  | [AT3G43470](http://www.arabidopsis.org/servlets/TairObject?name=AT3G43470&type=locus) | nucleic acid binding / zinc ion binding | Protein binding |
|  | [AT3G44070](http://www.arabidopsis.org/servlets/TairObject?name=AT3G44070&type=locus) | carbohydrate metabolic, glycoside hydrolase, family 35 | Carbohydrate metabolism |
|  | [AT3G10113](http://www.arabidopsis.org/servlets/TairObject?name=AT3G10113&type=locus) | myb family transcription factor | Transcription |
|  | [AT3G42860](http://www.arabidopsis.org/servlets/TairObject?name=AT3G42860&type=locus) | zinc knuckle (CCHC-type) family protein | Signalling |
|  | [AT4G08875](http://www.arabidopsis.org/servlets/TairObject?name=AT4G08875&type=locus) | Encodes a defensin-like (DEFL) family protein | Stress |
|  | [AT3G45470](http://www.arabidopsis.org/servlets/TairObject?name=AT3G45470&type=locus) | zinc finger protein-related | Protein binding |
|  | [AT3G28390](http://www.arabidopsis.org/servlets/TairObject?name=AT3G28390&type=locus) | P-GLYCOPROTEIN 18 (PGP18), ABC transporter | Transporter |
| **25_gene** | [AT3G43550](http://www.arabidopsis.org/servlets/TairObject?name=AT3G43550&type=locus) | carboxylesterase, hydrolase, lipase | Lipid biogenesis |
|  | [AT3G05930](http://www.arabidopsis.org/servlets/TairObject?name=AT3G05930&type=locus) | germin-like protein (GLP8) | Pathogen response |
|  | [AT3G22190](http://www.arabidopsis.org/servlets/TairObject?name=AT3G22190&type=locus) | IQ-domain 5 (IQD5), calmodulin binding | Signalling |
|  | [AT3G46510](http://www.arabidopsis.org/servlets/TairObject?name=AT3G46510&type=locus) | PLANT U-BOX 13 (PUB13) | Protein metabolism |
|  | [AT3G61350](http://www.arabidopsis.org/servlets/TairObject?name=AT3G61350&type=locus) | SKP1 interacting partner (SKIP4) | Protein metabolism |
|  | [AT4G05460](http://www.arabidopsis.org/servlets/TairObject?name=AT4G05460&type=locus) | F-box family protein (FBL20), ubiquitin-protein ligase | Protein metabolism |
|  | [AT3G07500](http://www.arabidopsis.org/servlets/TairObject?name=AT3G07500&type=locus) | far-red impaired responsive family protein / FAR1 family protein | Photosynthesis |
|  | [AT3G29774](http://www.arabidopsis.org/servlets/TairObject?name=AT3G29774&type=locus) | non-LTR retrotransposon family (LINE) | Transposon |
|  | [AT3G43723](http://www.arabidopsis.org/servlets/TairObject?name=AT3G43723&type=locus) | copia-like retrotransposon family | Transposon |
|  | [AT3G26380](http://www.arabidopsis.org/servlets/TairObject?name=AT3G26380&type=locus) | glycosyl hydrolase family protein 27, carbohydrate metabolic process | Carbohydrate metabolism |
|  | [AT2G23740](http://www.arabidopsis.org/servlets/TairObject?name=AT2G23740&type=locus) | SUVH6; methyl-CpG binding / methyl-CpNpG binding / methyl-CpNpN binding / methyltransferase | Histone modifications |
|  | [AT3G27550](http://www.arabidopsis.org/servlets/TairObject?name=AT3G27550&type=locus) | group II intron splicing factor CRS1-related | RNA splicing |
|  | [AT4G00450](http://www.arabidopsis.org/servlets/TairObject?name=AT4G00450&type=locus) | CRYPTIC PRECOCIOUS (CRP), mutant shows late flowering | Flowering |
|  | [AT4G04970](http://www.arabidopsis.org/servlets/TairObject?name=AT4G04970&type=locus) | GLUCAN SYNTHASE-LIKE 1 (GSL1) | Stress |
| **75_**  **promoter** | [AT4G04000](http://www.arabidopsis.org/servlets/TairObject?name=AT4G04000&type=locus) | transposable element gene; non-LTR retrotransposon family (LINE) | Transposon |
|  | [AT4G06513](http://www.arabidopsis.org/servlets/TairObject?name=AT4G06513&type=locus) | transposable element gene | Transposon |
|  | [AT3G22136](http://www.arabidopsis.org/servlets/TairObject?name=AT3G22136&type=locus) | putative non-LTR retroelement reverse transcriptase | Transposon |
|  | [AT4G06581](http://www.arabidopsis.org/servlets/TairObject?name=AT4G06581&type=locus) | gypsy-like retrotransposon family (Athila) | Transposon |
|  | [AT3G33377](http://www.arabidopsis.org/servlets/TairObject?name=AT3G33377&type=locus) | MuDR family transposase | Transposon |
|  | [AT4G08450](http://www.arabidopsis.org/servlets/TairObject?name=AT4G08450&type=locus) | disease resistance protein (TIR-NBS-LRR class) | Pathogen response |
|  | [AT4G08028](http://www.arabidopsis.org/servlets/TairObject?name=AT4G08028&type=locus) | Encodes a defensin-like (DEFL) family protein. | Stress |
|  | [AT4G08875](http://www.arabidopsis.org/servlets/TairObject?name=AT4G08875&type=locus) | Encodes a defensin-like (DEFL) family protein. | Stress |
|  | [AT3G32316](http://www.arabidopsis.org/servlets/TairObject?name=AT3G32316&type=locus) | AGAMOUS homolog | Flowering |
|  | [AT3G10113](http://www.arabidopsis.org/servlets/TairObject?name=AT3G10113&type=locus) | myb family transcription factor | Transcription |
|  | [AT3G44460](http://www.arabidopsis.org/servlets/TairObject?name=AT3G44460&type=locus) | basic leucine zipper transcription factor, DRBF2 | Transcription |
|  | [AT3G42860](http://www.arabidopsis.org/servlets/TairObject?name=AT3G42860&type=locus) | zinc knuckle (CCHC-type) family protein | Transcription |
|  | [AT4G09720](http://www.arabidopsis.org/servlets/TairObject?name=AT4G09720&type=locus) | ATRABG3A; Ras GTPase, intracellular protein transport, signal transduction | Signalling |
|  | [AT3G28390](http://www.arabidopsis.org/servlets/TairObject?name=AT3G28390&type=locus) | P-GLYCOPROTEIN 18 (PGP18), ABC transporter | Transporter |
| **75_gene** | [AT4G05585](http://www.arabidopsis.org/servlets/TairObject?name=AT4G05585&type=locus) | gypsy-like retrotransposon family | Transposon |
|  | [AT3G25815](http://www.arabidopsis.org/servlets/TairObject?name=AT3G25815&type=locus) | non-LTR retrotransposon family (LINE) | Transposon |
|  | [AT3G29620](http://www.arabidopsis.org/servlets/TairObject?name=AT3G29620&type=locus) | transposase IS4 family protein | Transposon |
|  | [AT4G03790](http://www.arabidopsis.org/servlets/TairObject?name=AT4G03790&type=locus) | gypsy-like retrotransposon family (Athila) | Transposon |
|  | [AT4G06631](http://www.arabidopsis.org/servlets/TairObject?name=AT4G06631&type=locus) | transposable element gene | Transposon |
|  | [AT3G07500](http://www.arabidopsis.org/servlets/TairObject?name=AT3G07500&type=locus) | far-red impaired responsive family protein, FAR1 | Flowering |
|  | [AT2G27350](http://www.arabidopsis.org/servlets/TairObject?name=AT2G27350&type=locus) | Ubiquitin-associated/translation elongation factor EF1B | Translation |
|  | [AT2G23740](http://www.arabidopsis.org/servlets/TairObject?name=AT2G23740&type=locus) | SUVH6; methyl-CpG binding / methyl-CpNpG binding / methyl-CpNpN binding / methyltransferase | Histone modifications |
|  | [AT3G25530](http://www.arabidopsis.org/servlets/TairObject?name=AT3G25530&type=locus) | gamma-hydroxybutyrate dehydrogenase (AtGHBDH), GLYOXYLATE REDUCTASE 1 (GLYR1) | Stress |
|  | [AT3G22190](http://www.arabidopsis.org/servlets/TairObject?name=AT3G22190&type=locus) | IQ-domain 5 (IQD5); FUNCTIONS IN: calmodulin binding | Signalling |
