# Supplementary material for: Transgenerational Adaptation of Arabidopsis to Stress Requires DNA Methylation and the Function of Dicer-Like Proteins
Source: PLoS One. 2010 Mar 3;5(3):e9514. doi: 10.1371/journal.pone.0009514 (PMC2831073; doi:10.1371/journal.pone.0009514)
Supplement: Table S3 — List of genes with over 80% hypomethylation either at promoter or transcribed regions of the S1_25 and S1_75 plants. (0.05 MB DOC) [file pone.0009514.s008.doc]

**Table S3. List of genes with over 80% hypomethylation either at promoter or transcribed regions of the S1_25 and S1_75 plants**

|  | **Locus ID** | **Gene Annotation** | **Possible role** |
| --- | --- | --- | --- |
| **25_**  **promoter** | [AT3G28030](http://www.arabidopsis.org/servlets/TairObject?name=AT3G28030&type=locus) | Repair of pyrimidine-pyrimidinone (6-4) dimmers, UVH3, UVR1 | DNA repair |
|  | [AT3G28020](http://www.arabidopsis.org/servlets/TairObject?name=AT3G28020&type=locus) | ATP binding / DNA binding | DNA binding |
|  | [AT3G28130](http://www.arabidopsis.org/servlets/TairObject?name=AT3G28130&type=locus) | nodulin MtN21 family protein | Transporter |
|  | [AT3G30350](http://www.arabidopsis.org/servlets/TairObject?name=AT3G30350&type=locus) | GOLVEN 3 (GLV3) | Signalling |
|  | [AT3G23110](http://www.arabidopsis.org/servlets/TairObject?name=AT3G23110&type=locus) | Receptor Like Protein 37 (AtRLP37) | Signalling |
|  | [AT4G03520](http://www.arabidopsis.org/servlets/TairObject?name=AT4G03520&type=locus) | chloroplast localized thioredoxin | Stress |
|  | [AT4G09550](http://www.arabidopsis.org/servlets/TairObject?name=AT4G09550&type=locus) | ATGCP3 INTERACTING PROTEIN 1 (GIP1) | Cell division |
|  | [AT3G45150](http://www.arabidopsis.org/servlets/TairObject?name=AT3G45150&type=locus) | TCP domain protein 16 (TCP16) | Transcription |
|  | [AT3G26618](http://www.arabidopsis.org/servlets/TairObject?name=AT3G26618&type=locus) | eukaryotic release factor 1-3 (ERF1-3) | Translation |
| **25_gene** | [AT3G28780](http://www.arabidopsis.org/servlets/TairObject?name=AT3G28780&type=locus) | functions in pollen exine formation | Pollen formation |
|  | [AT3G60565](http://www.arabidopsis.org/servlets/TairObject?name=AT3G60565&type=locus) | copia-like retrotransposon family, (AtRE1) | Transposon |
|  | [AT3G20170](http://www.arabidopsis.org/servlets/TairObject?name=AT3G20170&type=locus) | armadillo/beta-catenin repeat family protein | Signalling |
|  | [AT3G28450](http://www.arabidopsis.org/servlets/TairObject?name=AT3G28450&type=locus) | leucine-rich repeat transmembrane protein kinase | Signalling |
|  | [AT4G04740](http://www.arabidopsis.org/servlets/TairObject?name=AT4G04740&type=locus) | member of Calcium Dependent Protein Kinase | Signalling |
|  | [AT3G23120](http://www.arabidopsis.org/servlets/TairObject?name=AT3G23120&type=locus) | Receptor Like Protein 38 (AtRLP38) | Signalling |
|  | [AT3G48870](http://www.arabidopsis.org/servlets/TairObject?name=AT3G48870&type=locus) | Clpa regulatory subunit of CLP protease complex, ATCLPC | Chloroplast import |
|  | [AT2G32250](http://www.arabidopsis.org/servlets/TairObject?name=AT2G32250&type=locus) | FAR1-related sequence 2 (FRS2) | Photosynthesis |
| **75_**  **promoter** | [AT3G28030](http://www.arabidopsis.org/servlets/TairObject?name=AT3G28030&type=locus) | Repair of pyrimidine-pyrimidinone (6-4) dimmers, UVH3, UVR1 | DNA repair |
|  | [AT4G03520](http://www.arabidopsis.org/servlets/TairObject?name=AT4G03520&type=locus) | chloroplast localized thioredoxin, ATHM2 | Abiotic stress |
|  | [AT3G23110](http://www.arabidopsis.org/servlets/TairObject?name=AT3G23110&type=locus) | Receptor Like Protein 37 (AtRLP37) | Signalling |
| **75_gene** | [AT4G07874](http://www.arabidopsis.org/servlets/TairObject?name=AT4G07874&type=locus) | hAT-like transposase family (hobo/Ac/Tam3) | transposon |
|  | [AT3G23120](http://www.arabidopsis.org/servlets/TairObject?name=AT3G23120&type=locus) | Receptor Like Protein 38 (AtRLP38) | protein_coding |
