# Supplementary material for: Transgenerational Adaptation of Arabidopsis to Stress Requires DNA Methylation and the Function of Dicer-Like Proteins
Source: PLoS One. 2010 Mar 3;5(3):e9514. doi: 10.1371/journal.pone.0009514 (PMC2831073; doi:10.1371/journal.pone.0009514)
Supplement: Table S4 — “Fold” shows fold difference between S1_25 and C1 plants. (0.08 MB DOC) [file pone.0009514.s009.doc]

**Table S4. List of genes differentially expressed in S1_25 plants as compared to C1 plants**

| **Possible role** | **Gene annotation** | **Gene ID** | **Fold** | **P-value** |
| --- | --- | --- | --- | --- |
| Cytoskeleton | actin depolymerizing factor 1 - like protein | At4g34970 | 3.27 | 0.0005 |
| DNA repair | similar to DNA-3-methyladenine glycosylase I | At5g44680 | 4.12 | 0.0007 |
| Signalling | putative protein serine/threonine protein kinase ATPK10 | At5g01820 | 3.59 | 0.0062 |
| Stress | DRE CRT-binding protein DREB1C | At4g25470 | 6.88 | 0.0021 |
| Stress/hormones | DNA binding protein - like DNA binding protein EREBP-4 | At5g61600 | 5.08 | 0.0025 |
| Stress/hormones | like ethylene responsive element binding factor 5 | At5g07580 | 3.98 | 0.0022 |
| Development | GRAB1-like protein | At1g77450 | -3.28 | 0.0032 |
| DNA repair | 3-methyladenine DNA glycosylase, putative | At1g75230 | -3.88 | 0.0019 |
| DNA binding | putative C2H2-type zinc finger protein | At2g37430 | -4.17 | 0.0001 |
| Oxidative stress | peroxidase ATP3a | At5g64100 | -3.55 | 0.0045 |
| Oxidative stress | similar to anionic peroxidase | At1g14540 | -3.72 | 0.0020 |
| Oxidative stress | peroxidase C2 precursor like protein | At4g08770 | -3.91 | 0.0012 |
| Oxidative stress | putative glutathione S-transferase | At2g29440 | -4.33 | 0.0021 |
| Oxidative stress | peroxidase ATP24a | At5g39580 | -4.48 | 0.0020 |
| Oxidative stress | putative cytochrome P450 | At2g27690 | -9.73 | 0.0000 |
| Pathogen response | similar to alpha-hydroxynitrile lyase HNL4 | At5g10300 | -3.08 | 0.0067 |
| Pathogen response | disease resistance protein : TIR/NB-ARC/LRR | At1g56510 | -3.09 | 0.0017 |
| Pathogen response | xyloglucan endo-1,4-beta-D-glucanase-like protein | At4g30280 | -3.32 | 0.0043 |
| Pathogen response | disease resistance protein-like | At5g22690 | -3.50 | 0.0012 |
| Pathogen response | similar to peanut type II chitinase | At4g01700 | -3.80 | 0.0097 |
| Pathogen response | similar to disease resistance protein RPP1-WsA | At5g58120 | -3.83 | 0.0081 |
| Pathogen response | similar to harpin-induced protein hin1 | At2g35980 | -3.95 | 0.0080 |
| Pathogen response | thaumatin-like protein | At4g36010 | -4.15 | 0.0047 |
| Pathogen response | similar to Mlo proteins | At2g39200 | -5.92 | 0.0024 |
| Photosynthesis | NADPH-ferrihemoprotein reductase (ATR2) | At4g30210 | -3.26 | 0.0034 |
| Photosynthesis | putative monodehydroascorbate reductase (NADH) | At3g09940 | -7.01 | 0.0004 |
| Signalling | calcineurin B-like protein 1 | At4g17615 | -3.09 | 0.0081 |
| Signalling | serine/threonine kinase, putative | At1g01140 | -3.13 | 0.0003 |
| Signalling | wall-associated kinase 2, putative | At1g56120 | -3.23 | 0.0022 |
| Signalling | similar to protein Pto kinase interactor 1 | At3g59350 | -3.56 | 0.0022 |
| Signalling | similar to extra-large G-protein | At4g34390 | -3.57 | 0.0004 |
| Signalling | inositol 1,3,4-trisphosphate 5/6-kinase-like protein | At4g08170 | -3.63 | 0.0022 |
| Signalling | diacylglycerol kinase (ATDGK1) | At5g07920 | -3.86 | 0.0000 |
| Signalling | similar to receptor-like protein kinase 5 | At5g25930 | -3.92 | 0.0032 |
| Signalling | similar to receptor protein kinase (IRK1) | At1g61360 | -4.22 | 0.0079 |
| Stress response | putative anthocyanidin synthase | At2g38240 | -3.89 | 0.0061 |
| Stress response | similar to abscisic acid-induced protein HVA22 | At4g24960 | -4.45 | 0.0047 |
| Stress response | putative protein EREBP-3 homolog | At3g50260 | -4.58 | 0.0015 |
| Stress response | lipoxygenase | At1g17420 | -4.89 | 0.0018 |
| Stress response | low-temperature-induced protein 78 | At5g52310 | -7.53 | 0.0001 |
| Stress response | dehydrin Xero2 | At3g50970 | -27.59 | 0.0022 |
| Stress response | cold and ABA inducible protein kin1 | At5g15960 | -5.06 | 0.0061 |
| Stress/hormones | ethylene responsive element binding protein | At3g23230 | -3.43 | 0.0010 |
| Stress/hormones | putative jasmonic acid regulatory protein | At3g15500 | -3.78 | 0.0006 |
| Stress/hormones | auxin conjugate hydrolase (ILL5) | At1g51780 | -3.69 | 0.0030 |
| Transcription | RGA-like protein transcription factor | At5g17490 | -3.47 | 0.0005 |
| Transcription | TINY-like transcription factor | At1g22810 | -6.93 | 0.0002 |
| Transporter | plasma membrane-type calcium ATPase (ACA2) | At4g37640 | -3.08 | 0.0029 |
| Transporter | proton pump interactor | At4g27500 | -3.16 | 0.0020 |
